# Supplementary material for: Validation of the severe COVID-19 prognostic value of serum IL-6, IFN-λ3, CCL17, and calprotectin considering the timing of clinical need for prediction
Source: PLoS One. 2023 Mar 30;18(3):e0279897. doi: 10.1371/journal.pone.0279897 (PMC10062661; doi:10.1371/journal.pone.0279897)
Supplement: S1 Appendix — (DOCX) [file pone.0279897.s001.docx]

S1 Appendix. Biomarker measurement methods

*Measurement of calprotectin by* *absorption immunochromatography*

Quantum Blue® sCAL (BÜHLMANN Laboratories AG, Basel, Switzerland) was used as the measuring reagent. A Quantum Blue® Reader 2nd Generation was used as the measuring instrument. The procedure was performed as described in the instruction manual. Each serum sample was assayed three times. The average value was used as the final assay result.

*Measurement of calprotectin by* *an enzyme-linked immunosorbent assay (ELISA)*

A BÜHLMANN sCAL ELISA (BÜHLMANN Laboratories AG, Basel, Switzerland) was used as the assay reagent and performed as described in the instruction manual. An LSE Digital Microplate Shaker (CORNING Inc., NY, USA) was used to shake the ELISA plate, a SpectraMax iD5 (Molecular Devices, LLC, CA, USA) was used to measure absorbance, and PLATEmanager® software (TECAN group Ltd., Zurich, Switzerland) was used to convert absorbance to the concentration. Each serum sample was analyzed twice and the average value was used as the final result.

*Measurement of interferon-λ3 by a chemiluminescent enzyme immunoassay*

HISCL IFN-λ3 reagent (Sysmex Co., Ltd., Hyogo, Japan) was used as the assay reagent and an HISCL-5000 (Sysmex Co., Ltd. , Hyogo, Japan) as the assay device. The procedure was performed as described in the instruction manual. Sera frozen at 80°C were brought to room temperature and centrifuged at 1500 × g for 10 min to remove fibrin clumps before measurement.

*Measurement of TARC by a chemiluminescent enzyme immunoassay*

HISCL TARC reagent (Sysmex Co., Ltd., Hyogo, Japan) was used as the measuring reagent and the HISCL-5000 was used as the measuring device. The procedure was performed as described in the instruction manual.

*Measurement of IL-6 by an electrochemiluminescence immunoassay*

IL-6 was measured by the electrochemiluminescence immunoassay method using ECLusys reagent IL-6 (Roche Diagnostics K.K., Tokyo, Japan) as the measuring reagent and a Cobas e801 (Roche Diagnostics K.K., Tokyo, Japan) as the measurement device in accordance with the instruction manuals.
