# Supplementary figures and images for: Validation of the severe COVID-19 prognostic value of serum IL-6, IFN-λ3, CCL17, and calprotectin considering the timing of clinical need for prediction
Source: PLoS One. 2023 Mar 30;18(3):e0279897. doi: 10.1371/journal.pone.0279897 (PMC10062661; doi:10.1371/journal.pone.0279897)

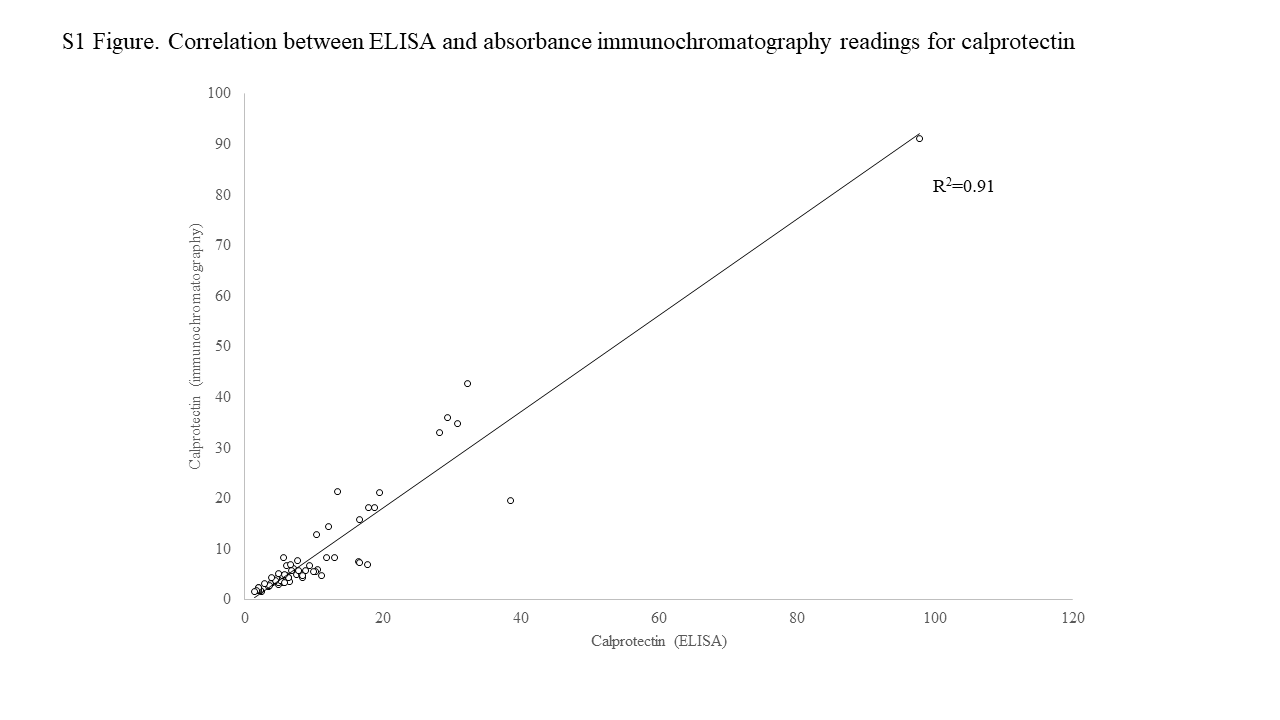

Supplement: S1 Fig — (TIF) [file pone.0279897.s002.tif]
