# Supplementary material for: Validation of the severe COVID-19 prognostic value of serum IL-6, IFN-λ3, CCL17, and calprotectin considering the timing of clinical need for prediction
Source: PLoS One. 2023 Mar 30;18(3):e0279897. doi: 10.1371/journal.pone.0279897 (PMC10062661; doi:10.1371/journal.pone.0279897)
Supplement: S1 Table — A: For prediction of future oxygen administration. B: For prediction of future mechanical ventilation administration or death. C: For prediction of future oxygen support with nasal high flow cannula or death. (DOCX) [file pone.0279897.s003.docx]

S1 Table. Multiple comparisons of the AUC of ROC curves (p-values)

1. For prediction of future oxygen administration

|  | IL-6 | IFNλ3 | TARC | Calprotectin |
| --- | --- | --- | --- | --- |
| IL6 | x | 0.271 | 0.458 | 0.209 |
| IFNλ3 | x | x | 0.0861 | 0.0764 |
| TARC | x | x | x | 1 |
| TARC: Thymus and activation-regulated chemokine | | | | |

B. For prediction of future mechanical ventilation administration or death

|  | IL-6 | IFNλ3 | TARC | Calprotectin |
| --- | --- | --- | --- | --- |
| IL-6 | x | 0.775 | 0.562 | 0.879 |
| IFNλ3 | x | x | 0.921 | 0.897 |
| TARC | x | x | x | 0.818 |
| TARC: Thymus and activation-regulated chemokine | | | | |

C. For prediction of future oxygen support with nasal high flow cannula or death

|  | IL-6 | IFNλ3 | TARC | Calprotectin |
| --- | --- | --- | --- | --- |
| IL-6 | x | 0.085 | 0.563 | 0.91 |
| IFNλ3 | x | x | 0.279 | 0.0946 |
| TARC | x | x | x | 0.624 |
| TARC: Thymus and activation-regulated chemokine | | | | |
