# Supplementary material for: Validation of the severe COVID-19 prognostic value of serum IL-6, IFN-λ3, CCL17, and calprotectin considering the timing of clinical need for prediction
Source: PLoS One. 2023 Mar 30;18(3):e0279897. doi: 10.1371/journal.pone.0279897 (PMC10062661; doi:10.1371/journal.pone.0279897)
Supplement: Attachment — Submitted filename: Response_to_reviwers1.0.docx [file pone.0279897.s004.docx]

Reviewer #1:

*Dear authors*

*Thanks for the interesting study.*

*The study design was impressed and valuable, but with major limitation, such as too small size.*

*Otherwise, beside study population size, I suggest that you could add outcome study in the future.*

Thank you for your comments. We are aware that the sample size is a major limitation. However, during the review process, another research group, to which some of the coauthors belong, published a study with a slightly larger sample size (Suzuki T, et al. Eur J Clin Invest 2022), which showed data that supported the “Prediction A” results for IFNλ3 and TARC. Therefore, we have included this in the “Limitations” section. However, there were only eight patients who required advanced respiratory support beyond high-flow oxygen therapy in this other study and they were not receiving oxygen. Therefore, “Prediction B” still needs to be validated.

As suggested by the reviewer, we have added a perspective on outcome studies to determine whether patients were able to continue without hospitalization using the biomarkers.

In response to these points, the " However, for IFN-λ3 and TARC, a similar trend to the present study was observed in a larger sample size validation of a report that predicted the occurrence of future oxygen demand (Prediction A) in the early disease stages when oxygen administration had not started [40]. Prediction B, which predicts whether patients on oxygen will become more severely ill, was not demonstrated in this other report [40], and future studies should include this prediction. Outcome studies are also expected to reveal how many patients actually avoid hospitalization using biomarkers." was added in the Limitations section.

Revisions are highlighted in yellow in the text.

Reviewer #2:

*The authors submitted a research article in which they examined the ability of four biomarkers to predict disease severity using conserved sera collected at the appropriate time for prediction. They enrolled 18 and 45 hospitalized patients aged 20 years or older in study 1 and 2, respectively. Since then they collected blood samples, measured biomarkers, calculated the PREDI-CO score by the severity prediction model extracted from the above data and the simple risk score by the oxygen administration prediction model created from registry data in Japan. The authors found that the prediction of mechanical ventilator management was similar for IL-6, IFN-λ3, TARC, and CP biomarkers, and the PREDI-CO score. They concluded that there were no any biomarker that showed a significant predictive ability. The aim of the study is clear and concise. The manuscript has a logical structure and well-balanced subsections that cover all aspects of the initial hypothesis. The tables and figures are clear and legible. The conclusive part seems to show informative description of the finding significance. Although the study has a small sample size and retrospective design, it appears to be intriguing, because it confirms a role of conventional score to stratify the patients at risk. However, I would like to put forward several comments to discuss.*

Thank you for your comments. We have addressed your comments below.

Revisions are highlighted in yellow in the text.

1. *Whether the authors pooled the biomarkers fully related to all aspects of the natural evolution of the disease?*

Although only some biomarkers were examined in this study, they are representative biomarkers that have been suggested to be involved in disease exacerbation in previous studies. However, direct comparisons with LDH and lymphocytes, which are common biomarkers associated with COVID-19 prognosis, are not possible. However, because these biomarkers are included in the PREDI-CO score, although the timing of evaluation is simplified to the time of admission, we believe that this is an indirect evaluation.

2. *Is it reasonable to identify different biomarkers at different pathological stages of the disease?*

Changing the biomarkers to be measured in detail for each stage of the pathological condition may not be practical in terms of some aspects, considering the cost and management complexity involved in the testing system. However, if the purpose of the medical facility is clear, we believe it is worthwhile. For example, predicting future oxygenation disorders in a clinic that mainly provides initial medical care or predicting more serious conditions (e.g., high-flow oxygen therapy and ventilatory management) in a hospital that does not provide advanced medical care.

3. *Please, give extensive explanation of the main reason why the biomarker model was not became effective.*

Although we cannot draw definite conclusions at this stage, a study with a slightly larger sample size from another research group, to which some of the coauthors belong, showed data that support the “Prediction A” results for IFN-λ3 and TARC. It is presumed that IFN-λ3 has a high predictive ability for future oxygen demand in the early disease stages when oxygen may not be required. However, when more severe conditions are observed, i.e., when intubation and ventilation are required, a combination of exacerbating factors may occur, such as a secondary bacterial infection and cytokine storm. Therefore, we believe that each biomarker may have similar predictive performance. Therefore, the prediction of disease severity using more than one biomarker had a higher AUC. Although it was not the primary outcome of this study, we believe that the use of multiple biomarkers should be considered when predicting recurrence of severe disease. We have included some discussion of these considerations in the Discussion section.
