# Supplementary material for: Validation of the severe COVID-19 prognostic value of serum IL-6, IFN-λ3, CCL17, and calprotectin considering the timing of clinical need for prediction
Source: PLoS One. 2023 Mar 30;18(3):e0279897. doi: 10.1371/journal.pone.0279897 (PMC10062661; doi:10.1371/journal.pone.0279897)
Supplement: Attachment — Submitted filename: Response_to_reviwers2.1.docx [file pone.0279897.s005.docx]

Reviewer #1:

*Thanks for the authors' comprehensive response, I do not have any more questions!*

*I would like to suggest this manuscript be accepted for publication.*

**Thank you for your constructive comments.**

Reviewer #3:

*The main aim/hypothesis of the draft paper is really crucial in our clinical practice.*

*However, I have major concerns listed below.*

**Thank you for your useful comments.**

**Our point–by-point responses are below. Revisions are highlighted in yellow.**

Abstract section:

*1.“…collected at the appropriate time…”. What do the authors mean? They should be clear about appropriate time.*

**Regarding the appropriate timing, the following text was added: “****Here, we examined the ability of four biomarkers to predict disease severity using conserved sera from COVID-19 patients who received inpatient care between January 1, 2020 and September 21, 2021 at the National Center for Global Health and Medicine, collected at the appropriate time for prediction. We predicted illness severity in two situations: 1)** **prediction of future oxygen administration for patients without oxygen support within 8 days of onset (Study 1) and** **2) prediction of future mechanical ventilation support (excluding non-invasive positive pressure ventilation) or death of patients within 4 days of the start of oxygen administration (Study 2).”**

*2.Statistical methods might be excluded from the abstract section. They should be stated in the text (Method section). Instead, the method (sampling time, etc) or the features of the cases should be stated in abstract. Understanding of the methodology is difficult from the abstract section. For instance, current study cases have COVID pneumonia or COVID-associated other organ involvements??? The definitions of prediction A and B are not clear as in Introduction part of the text.*

**Thank you for your comments. Although reviewer #3 recommended omitting the description of statistical analysis, we retained the first sentence “AUC was calculated and compared by ROC curves” because we believed that deleting it would make the results unclear.**

**In the Abstract, “Prediction A” and “Prediction B” were changed to “Study 1” and “Study 2”,** **respectively, to align with the main text. Research methods have been added, including the study duration, methods to obtain clinical information, and comparison of the predictive ability.**

**The sentence “2) prediction of future oxygen administration within 4 days of the start of oxygen administration (excluding non-invasive positive pressure ventilation; prediction B)” was changed to “2) prediction of future mechanical ventilation support (excluding non-invasive positive pressure ventilation) or death of patients within 4 days of the start of oxygen administration (Study 2)” because it incorrectly stated what was to be considered.**

**“****Study 1 included 18 patients, five of whom had developed oxygen needs. Study 2 included 45 patients, 13 of whom required ventilator management or died” was also added to the manuscript. However, this report is not a case study and we believe that detailed patient features are not needed in the abstract.**

*3. In conclusion part of the abstract, authors stated a sentence as “….combination of biomarkers was predictive of mechanical ventilator requirements…..”. There was no any result to conclude this in result section of the abstract. Those results should be added as “Which combinations? 2 or 3 combinations?.. etc”.*

**The “the number of” statement appears to have been lost and has been corrected. Additionally, the sensitivity and specificity when two or more tests were positive have been described and changed as follows: “The number of biomarkers above the cutoff showed the possibility of good prediction with an AUC of 0.86 (95% CI 0.75–0.97). When two or more biomarkers were positive, sensitivity and specificity were 0.92 and 0.63, respectively. In terms of biomarker testing at times when prognostication may be clinically useful, IFN-λ3 was predictive of oxygenation demand and a combination of four biomarkers was predictive of mechanical ventilator requirement.”**

*4.In conclusion part of the abstract, small sample size is limitation of the text. It should be in the text as limitations.*

**We have removed this part of the text in response to your suggestion.**

Method section:

*1. They should explain the components of PREDI-CO score. How do they define severity as high or low?*

**PREDI-CO predicts severe respiratory failure and we have added the definition. We have also included the “high risk” definition to address the later point. “The PREDI-CO score predicts severe respiratory failure, which is defined by WHO criteria SpO_2_ <93% with 100% FiO_2_ (reservoir mask, continuous positive airway pressure ventilation, or other non-invasive ventilation), respiratory rate >30 breaths/minute, or respiratory distress. Three or higher predicts a high risk for severe disease.”**

*2.In Table 1. The “high risk” mean is not clear according to the result section?*

**We have added a note to the Methods regarding the fact that scoring was performed in accordance with the age group.**

*3.For “…median values of IL-6, IFN-λ3, TARC, and CP were 10.4 (4.0–20.9)…” The ranges show us (min-max) OR (IQR). As far as I understood it shows us IQR; however, it should be stated again in here.*

**In the statistical analysis section, we stated that “Discrete and continuous data are expressed as numbers (percentages) and medians [interquartile range (IQR)], respectively,” but we have added that all of them are IQR. In some places, 95% CI was used. Therefore, we have specified this clearly.** **The term "Discrete and continuous data" has been changed to "****Qualitative and quantitative data" because the terminology was not accurate.**

*4. There is confusion about Table numbers. I think, they mean Table 2, while they are stating Table 3 in the text.*

**Thank you for pointing this out. We have corrected the citation to Table 2.**

*5.For the cases included in Study 1 and Study 2, there might be a Flow chart (Figure x) to understand actual situation. For example, I could not understand whether 5 cases required ECMO are needed non-invasive positive pressure ventilation OR invasive mechanical ventilator OR NOT. Via flow chart, understanding will be easy.*

**As reviewer #3 indicated, we have included a flowchart diagram summarizing the outcomes as Fig. 2.**

*6.There is no any statement for Figure 1 in the text.*

**The description has been included in the “Introduction.”**

*7.The legend of Figure 2A and 2B should be detailed. It is not easily understandable in this way. Moreover, supplement figure 2 and figure 3 might be added to Figure 2 as 2C and 2D.*

**Supplementary Figure 3 has been integrated as Fig. 3C and an explanation was added. If you still believe that the explanation is insufficient, we would appreciate it if you could point out specific areas that need to be explained. However, because Supplementary Figure 2 is difficult to integrate because of its title, we have added it as Fig. 4.**

*8.For ROC curves, while they are writing “significant”, is it possible to show “p values”?*

**The statistical analysis software EZR used does not calculate a p-value when calculating the AUC. We believe that whether the lower limit of the 95% confidence interval is greater than 0.5 is sufficient to determine whether the result is significant. However, using SPSS, the p-value can be calculated as asymptotic significance (two-tailed). The following table shows the results.**

| **p value** | **Calprotectin** | **IFNλ3** | **IL-6** | **TARC** | **CRP** |
| --- | --- | --- | --- | --- | --- |
| **Study 1** | **0.59** | **0.008** | **0.094** | **0.59** | **0.70** |
| **Study 2**  **To predict future mechanical ventilation administration or death** | **0.017** | **0.016** | **0.040** | **0.011** | **0.057** |
| **To predict future oxygen support above a nasal high flow cannula** | **0.239** | **0.002** | **0.19** | **0.056** | **0.46** |

**However, the sentence “A significant difference was observed only for IFN-λ3” was deleted because it was redundant. Additionally, the p-value for the comparison of medians was added as follows “with significant differences only for IFN-λ3 (p=0.0017).”**

*9.Exact number of the cases treated with anti-IL 6 or other immunomodulatory should be stated in the text.*

**There were three patients and the following statement was added to the text: “Two patients were receiving an anti-IL-6 antibody (tocilizumab) and one patient was receiving a JAK inhibitor at the time of serum sampling.”**

*10.I could not see the mortality ratios for the cases included in Study 1 and 2. Should be stated.*

**They were no mortality in Study 1 and 15.6% (7/45) in Study 2, which was already listed in Study 2, but the numbers were incorrect, which have been corrected and the percentages added.**

*They should state the implementation need of a biomarker such as IFN-λ3.*

**Because there are many limitations and the disease severity changes drastically, we could not conclude from the results that the introduction of biomarkers should be emphasized. However, the utility of such a biomarker, if identified, was described in the “Introduction” section.**
